# Supplementary material for: Comparative genomics provides new insights into the diversity, physiology, and sexuality of the only industrially exploited tremellomycete: Phaffia rhodozyma
Source: BMC Genomics. 2016 Nov 9;17:901. doi: 10.1186/s12864-016-3244-7 (PMC5103461; doi:10.1186/s12864-016-3244-7)
Supplement: Additional file 6: — List of orphan genes with links to PFAM (related to Additional file 1: Table S1). (ZIP 1428 kb) [file 12864_2016_3244_MOESM6_ESM.zip › PFAM_UNIQUE/G00192_P.html]

#                                                                            --- full sequence --- -------------- this domain -------------   hmm coord   ali coord   env coord
  
# target name        accession   tlen query name           accession   qlen   E-value  score  bias   #  of  c-Evalue  i-Evalue  score  bias  from    to  from    to  from    to  acc description of target
  
#------------------- ---------- ----- -------------------- ---------- ----- --------- ------ ----- --- --- --------- --------- ------ ----- ----- ----- ----- ----- ----- ----- ---- ---------------------
  
G00192\_P             -            412 Selenoprotein\_S      PF06936.6    190     0.017   13.3   3.8   1   1   3.9e-05     0.033   12.4   3.8    68   128   105   164    99   186 0.80 - Selenoprotein S (SelS)  
G00192\_P             -            412 Vps5                 PF09325.5    236       1.7    6.5   6.6   1   1    0.0063       2.7    5.8   6.6   142   183   125   166   116   170 0.92 - Vps5 C terminal like
